# Draft Genome Sequence of Two Monosporidial Lines of the Karnal Bunt Fungus *Tilletia indica* Mitra (PSWKBGH-1 and PSWKBGH-2)

**DOI:** 10.1128/genomeA.00928-16

**Published:** 2016-09-15

**Authors:** Pradeep Sharma, Ratan Tiwari, M. S. Saharan, Indu Sharma, Jitender Kumar, Shefali Mishra, Senthilkumar K. Muthusamy, R. K. Gupta, Sarika Jaiswal, M. A. Iquebal, U. B. Angadi, Neeraj Kumar, Samar Fatma, Anil Rai, Dinesh Kumar

**Affiliations:** a Crop Improvement Division, ICAR-Indian Institute of Wheat and Barley Research, Karnal, India; b Crop Protection Division, ICAR-Indian Institute of Wheat and Barley Research, Karnal, India; c Quality and Basic Science Division, ICAR-Indian Institute of Wheat and Barley Research, Karnal, India; d Centre for Agricultural Bioinformatics, ICAR-Indian Agricultural Statistics Research Institute, New Delhi, India

## Abstract

Karnal bunt disease caused by the fungus *Tilletia indica* Mitra is a serious concern due to strict quarantines affecting international trade of wheat. We announce here the first draft assembly of two monosporidial lines, PSWKBGH-1 and -2, of this fungus, having approximate sizes of 37.46 and 37.21 Mbp, respectively.

## GENOME ANNOUNCEMENT

Karnal bunt (KB) is named after the place Karnal in India, where it was first discovered in wheat crops in 1931 (1). It is reported that the loss in yield due to Karnal bunt is insignificant, i.e., only 0.3 to 0.5%, even under epidemic conditions (2). Although it does not actually result in devastating crop loss, it has full potential to decrease yield and actually hampers economy due to the quarantine regulations, i.e., debarring the export of KB-infected wheat grains. Karnal bunt disease is caused by the smut fungus *Tilletia indica* (syn. *Neovossia indica* [Mitra] Mundkur) a basidiomycetes belonging to the subdivision *Ustilaginomycotina*. In nature, *T. indica* exists primarily as teliospores, which are widespread in the seeds of the host plant inside or outside and soil. Under weather conditions of relative humidity over 70%, daytime temperatures between 18 and 24°C, and soil temperatures ranging from 17 to 21°C, the severity of this disease is increased (3). KB is identified in almost all the major wheat-producing regions of India, Iraq, Nepal, Pakistan, Afghanistan, the United States, Australia, and South Africa (4). Few sources of resistance have been reported (5). KB-infected wheat flour is not fit for human consumption due to its foul smell and taste if the infections in grains exceed 3% (6).

The Karnal bunt fungus *T. indica* samples (PSWKBGH-1 and PSWKBGH-2) were isolated from naturally infected wheat grain in Karnal in 2013. The genomic DNA was randomly interrupted and used to construct a pUC18 plasmid with a 180-bp inserted sequence. Sequencing platform technologies, i.e., Illumina NextSeq 500 PE (2 × 150 bp) and PacBio SMART hybrid sequencing technologies were used. The reads were quality checked using FastQC (7). The low-quality bases and adapter trimming were performed using in-house Perl scripts.

*De novo* genome assembly was performed using SPAdes version 3.5.0 (8). Assembled contigs were further scaffolded using the SSPACE program (9) using Illumina reads. A total of 366 and 470 scaffolds were generated from PSWKBGH-1 and PSWKBGH-2, respectively, with genome sizes of 37,460,344 bp (*N*_50_, 200,513 bp) and 37,216,861 bp (*N*_50_, 132,740 bp), respectively.

For comprehensive annotation of the genome, along with pathway identification and simple sequence repeat (SSR) marker discovery, scaffold sequences annotation was carried out using AUGUSTUS, Blast2GO Pro version 3.3 (10), KASS (11), and MISA (12). A total of 12,046 and 12,129 protein-coding gene models were predicted for PSWKBGH-1 and PSWKBGH-2, respectively, using AUGUSTUS. GO result showed that maximum values of ATP binding, DNA integration, and integral arrangement of membranes were associated with molecular function, biological processes, and cellular components, respectively. Also, 10,125 and 9,952 putative SSR markers were identified in PSWKBGH-1 and PSWKBGH-2, respectively. This is the first genome sequence of a fungus in the order *Georgefisheriales* (*Exobasidiomycetes*).

This work will provide baseline data to understand key genes controlling mating and virulence factors, deciphering information related to destructive pathogen. The mechanisms of the wheat-infecting pathogen might provide knowledge on its molecular identification and management of Karnal bunt disease. This would also be pivotal in protecting wheat grain quality.

### Accession number(s).

The present draft genome assembly versions have been deposited at GenBank under the accession numbers MAPW00000000 (GenBank assembly accession: GCA_001689995.1) and MAPX00000000 (GenBank assembly accession: GCA_001689945.1) for PSWKBGH-1, and PSWKBGH-2, respectively.
